# Source Identification and Apportionment of Potential Toxic Elements in Soils in an Eastern Industrial City, China

**DOI:** 10.3390/ijerph19106132

**Published:** 2022-05-18

**Authors:** Feng Li, Mingtao Xiang, Shiying Yu, Fang Xia, Yan Li, Zhou Shi

**Affiliations:** 1 College of Materials and Environmental Engineering, Hangzhou Dianzi University, Hangzhou 310018, China; lifeng@hdu.edu.cn; 2 Institute of Land Science and Property, School of Public Affairs, Zhejiang University, Hangzhou 310058, China; xiangmt@zju.edu.cn (M.X.); 15968172022@163.com (S.Y.); liyan522@zju.edu.cn (Y.L.); 3 College of Economics and Management, Zhejiang A&F University, Hangzhou 311300, China; 4 Key Laboratory of Environment Remediation and Ecological Health, Ministry of Education, Hangzhou 310058, China; shizhou@zju.edu.cn; 5 College of Environmental and Resource Sciences, Zhejiang University, Hangzhou 310058, China

**Keywords:** potential toxic elements, source identification, source apportionment, PMF, Unmix, industrial city

## Abstract

The extensive pattern of economic growth has an inestimable negative impact on the ecological environment, which causes the soil pollution problem to become increasingly prominent. In order to improve the effectiveness and rationality of prevention and control of heavy metal pollution in regional soil, it is necessary to understand the current situation of pollution, identify pollution sources and clarify future pollution risks. In this paper, an industrially developed city in eastern China was taken as the study region. The positive matrix factorization model (PMF) model and Unmix model was applied to identify and apportion the pollution sources of soil potential toxic elements after evaluating the ecological risk of soil potential toxic elements. The PMF model identified six factors, including single source and composite source. The Unmix model also identified six sources, including sources of nature, industrial discharge and traffic emissions. The comparison between the two models showed that Hg and Ni pollution, as well as Cr enrichment in the study region, were related to the industrial discharge from enterprises and factories. Cd pollution was related to traffic emission sources. Cu and Zn pollution were related to the multiple sources mixed with soil parent material, traffic emissions and industrial discharge from electronic enterprises. Pb pollution was related to natural sources (e.g., soil pH) but also to industrial sources (e.g., industrial wastes discharge). Enrichment was related to soil parent material and agricultural inputs. Our study also implies that soil heavy metal pollution or enrichment in the study region was mainly from anthropogenic sources and supplemented by natural sources.

## 1. Introduction

The rapid progress of industrialization and urbanization is not completely divorced from extensive development models of the past [1,2]. The irrational industrial structure and the lack of technology for sustainable development have led to the total discharge of pollutants remaining high [3]. The soil pollution continues to expand and deepen, which seriously damages the safety of agricultural products and human health [4,5,6]. Potential toxic elements in soil, in particular, have cumulative effects of teratogenic, cancer and mutation [7]. When the amount of potential toxic element accumulation reaches up to a certain extent, potential toxic elements may be suddenly activated, posing a serious threat on food safety and human living environments [8,9]. Furthermore, since the potential toxic elements in soil are easy to accumulate and latent but difficult to remove [10], it is more complicated and time-consuming to restore contaminated soil than gas and water [11,12,13]. Therefore, cutting off soil pollution pathways as far as possible, as well as strengthening soil potential toxic element monitoring and control, is of vital significance to curb the deterioration trend of soil pollution and protect the soil ecological environment [6,14].

Analyzing and identifying the sources of potential toxic elements in soil is necessary to make decisions for subsequent prevention and management of soil pollution [15]. The sources of potential toxic elements in soil are mainly divided into natural sources (mainly parent material and soil forming process) [16,17] and human sources (such as industrial discharges, automobile exhausts, fertilizer and pesticide inputs, agricultural film application and sewage irrigation) [3,18,19,20]. The latter sources lay a great influence on the contents of potential toxic elements in soil. At present, the source apportionment models of soil potential toxic elements also have two categories. The one is a diffusion model that takes pollution sources as the research object, and which depends on the pollutant emission inventory for calculation. The other is a receptor model that takes contaminated areas as the research object, such as chemical mass balance (CMB), factor analysis (FA), principal component analysis (PCA), positive matrix factorization analysis (PMF), Unmix model, cluster analysis, projection pursuit regression and genetic algorithm [4,5,15,21]. In recent years, a growing number of research has focused on the source apportionment of soil potential toxic elements with popular methods of isotope tracer, such as the PMF model, UNMIX model and PCA model. In particular, the PMF model and UNMIX model are both proposed receptor models based on the principle of factor analysis and improved based on the PCA model, and which are recommended by the United States Environmental Protection Agency (USEPA). Although the PMF model and UNMIX model have been widely used in previous studies [22,23,24,25], the single analytical method for sources apportionment has certain limitations. Owing to the inevitable systematic error and sample influence generated by the algorithm, the diversified integration and comprehensive application of various source apportionment methods have distinct advantages for exploring pollution sources of soil potential toxic elements [26,27].

In this study, an industrial city in Southeast China was selected as a case study region, and the PMF and Unmix models were applied to identify and apportion the pollution sources of potential toxic elements in soils. The specific aims were to: (1) evaluate the pollution level of potential toxic elements in soil through ecological risk assessment; (2) identify and apportion the pollution sources of potential toxic elements in soil based on the PMF and Unmix models. The results would provide significant information to verify the quantitative sources as well as restore and manage the contaminated soil in industrial areas in the future.

## 2. Materials and Methods

### 2.1. Study Region

**Figure 1:**
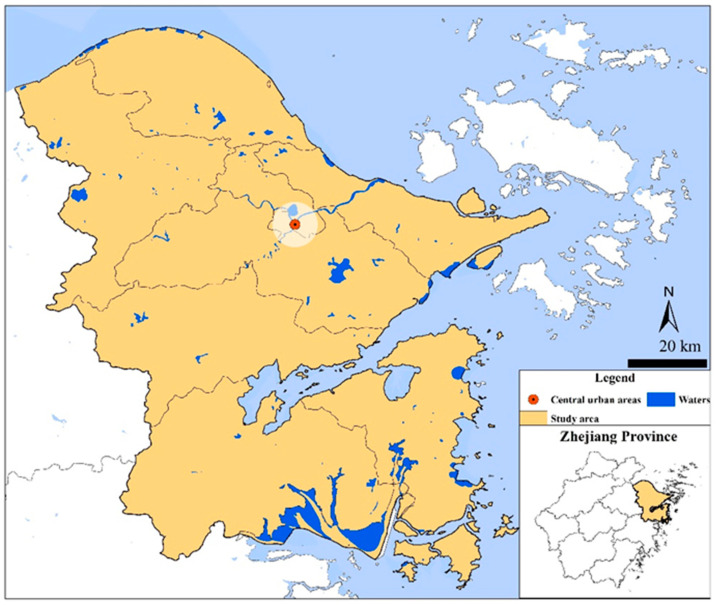
Location of the study region.

A coastal city in eastern Zhejiang Province was selected as the study region, which is located in the middle of China’s coastline (Figure 1). The elevation gradually decreases from southwest to northeast, and the plain is the main landform (40.3%). The study region belongs to the northern subtropical monsoon climate with suitable precipitation that contributes to local developed agriculture [7]. Despite the fertile edaphic condition, soil acidification is widespread. Most of the soil was acidic (pH < 6.5), and some even showed a strong acidity (pH < 5.5). Moreover, as an important economic center of the province, the city has dense road networks and well-developed industries. In particular, the automobile industry, electrical machinery and equipment industry, chemical raw materials and products industry and fuel processing industry make the largest contributions. In addition, chemical metallurgy enterprises, mechanical electronics enterprises, textile enterprises and mining enterprises are four primary types of industrial enterprises in the study region [28].

### 2.2. Data

**Figure 2:**
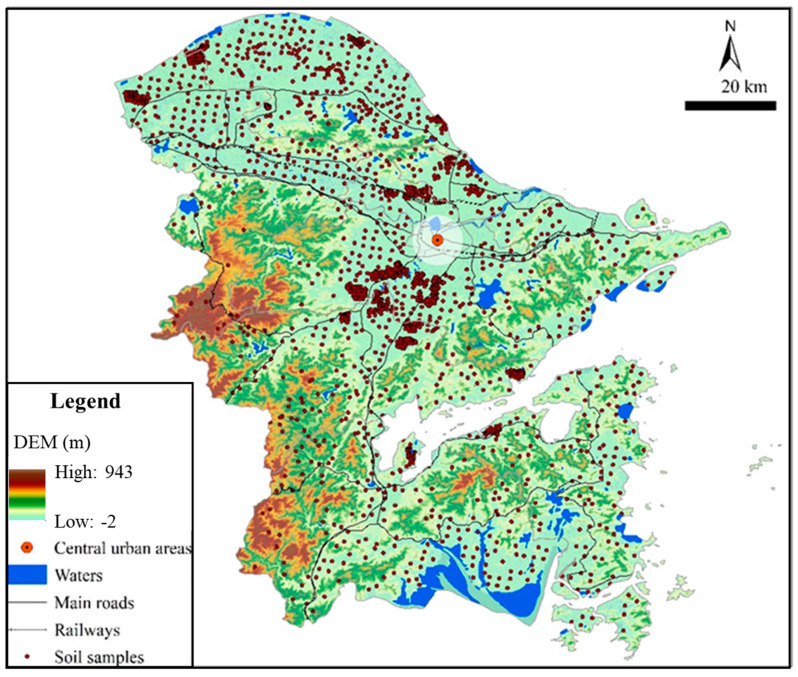
The distribution of soil potential toxic element samples in the study region.

Soil samples were from the survey project of soil heavy metal pollution in Zhejiang Province in 2013. A total of 2051 soil samples of agricultural land surface (0–20 cm) were collected in the study region (Figure 2). The sampling sites were arranged with the method of uniform distribution, and the soil samples were collected by the plum blossom sampling method. According to the agricultural sector standard (NY/T 1377-2007) of the People’s Republic of China, soil pH was measured in H_2_O with a soil/solution ratio of 1:2.5 (*m/v*) using the Glass Electrode method (GL, pHS-3C, REX, Shanghai, China). Hg was determined by the double channel atomic fluorescence spectrometer after being digested by HNO_3_-HCl in a water bath. Cr, Pb, As, Ni, Zn and Cu were determined by the inductively coupled plasma optical emission spectrometry (ICP-OES 6300, Thermo Fisher Scientific, Waltham, MA, USA) after being acid-digested with HCl-HNO_3_-HClO_4_. Cd were determined by the inductively coupled plasma mass spectrometer (ICP-MS, Agilent 7500a, Palo Alto, CA, USA) after being digested by HF-HNO_3_-HClO_4_. In order to ensure the accuracy and reliability of determination results, parallel, blank and standard reference materials were used for quality assurance and quality control [1,7].

### 2.3. Methods

#### 2.3.1. Ecological Risk Assessment

An ecological risk index was proposed by Hakanson [29], which can quantitatively reflect not only the impact of each potential toxic element in a specific environment, but also the comprehensive impact of multiple potential toxic elements in the environment. It can be expressed by the following formulas:

(1) $$E_{r}^{i}=T_{r}^{i}\times \frac{C_{r}^{i}}{C_{0}^{i}}$$

(2) $$RI=\sum_{i=1}^{m}E_{r}^{i}\;$$

where: $C_{r}^{i}$ represents the measured concentration of the *i*th potential toxic element at the *r*th sampling location. $C_{0}^{i}$ represents the soil environmental quality standard of *i*th potential toxic element, which takes the soil background values of Zhejiang province as standards, i.e., As (9.2 mg/kg), Hg (0.086 mg/kg), Cr (52.9 mg/kg), Cd (0.07 mg/kg), Pb (23.7 mg/kg), Cu (17.6 mg/kg), Zn (70.6 mg/kg) and Ni (24.6 mg/kg) [7]. $T_{r}^{i}$ represents the toxicity response parameter of the *i*th potential toxic element at the *r*th sampling location, which reflects the toxicity level of potential toxic elements and the sensitivity of organisms to heavy metal pollution, i.e., As (10), Hg (40), Cr (2), Cd (30), Pb (5), Cu (5), Zn (1) and Ni (5) [7,29]. $E_{r}^{i}$ is the ecological risk index of a certain type of potential toxic element, which can be divided into five grades, i.e., Slight risk ($E_{r}^{i}\;$ < 40), Mild risk (40 ≤ $\;E_{r}^{i}\;$< 80), Moderate risk (80 ≤ $E_{r}^{i}$ < 160), Severe risk (160 ≤ $E_{r}^{i}$ < 320) and Extremely severe risk ($E_{r}^{i}$ ≥ 320). *RI* is the comprehensive ecological risk index, which can be divided into four grades, i.e., Slight risk (*RI* < 150), Mild risk (150 ≤ *RI* < 300), Moderate risk (300 ≤ *RI* < 600) and Severe risk (*RI* ≥ 600).

#### 2.3.2. Positive Matrix Factorization Model

The positive matrix factorization (PMF) model is an improved factor analysis receptor model developed by Paatero and Tappert [30]. Since the source component spectrum and source contribution rate obtained by PMF model have explicable and definite physical significance compared with other traditional factor analysis models, the PMF model has been successfully applied to the source analysis of environmental pollutants. In this model, the original matrix composed of receptor sample concentration data can be decomposed into a factor score matrix, a factor load matrix and a residual matrix [31]. The basic matrix is as follows:

(3) $$X_{ij}=\sum_{k=1}^{p}G_{ik}F_{kj}+E_{ij}$$

where: $X_{ij}$ represents the concentration of the *j*th potential toxic element of the *i*th sample, $F_{kj}$ represents the concentration of the *j*th potential toxic element of source *k* (i.e., the spectral matrix of source component), $G_{ik}$ represents the contribution of source *k* to the *i*th sample, (i.e., the source contribution matrix), $E_{ij}$ represents the residual matrix of the *j*th potential toxic element of the *i*th sample and *p* is the number of main factors (i.e., the number of main sources).

The PMF model is iteratively calculated based on Multilinear engine 2 algorithm with the original matrix continuously decomposed to obtain the optimal factor score matrix and factor load matrix, in order to achieve optimization by minimizing the objective function Q [32]. The formula is as follows:

(4) $$Q=\sum_{i=1}^{n}\sum_{j=1}^{m}{\left(\frac{E_{ij}}{U_{ij}}\right)}^{2}$$

where: *n* is the number of receptor samples, *m* is the types of measured potential toxic elements and $U_{ij}$ represents the uncertainty of the *j*th potential toxic element of the *i*th sample. This uncertainty is used to process the weight of each individual sample, which is calculated as [33]:

(5) $$U=\{\begin{array}{l} \sqrt{{\left(EF\times C\right)}^{2}+{\left(MDL\times 0.5\right)}^{2}},C>MDL\\ \frac{5}{6}\times MDL,C\leq MDL \end{array}$$

where: *EF* is the error fraction, *C* is the measured concentration of potential toxic element samples and *MDL* represents the Method Detection Limit that is determined by the detecting instrument. For the samples in this study, the *MDL* of As, Hg, Cr, Cd, Pb, Cu, Zn and Ni was 0.01 mg/kg, 0.005 mg/kg, 5 mg/kg, 0.05 mg/kg, 0.2 mg/kg, 1 mg/kg, 0.5 mg/kg and 5 mg/kg, respectively.

#### 2.3.3. Unmix Model

The Unmix model is another receptor model proposed by the US Environmental Protection Agency to solve general environmental problems [34]. This model takes the concentrations of samples as parameters, which can avoid the normalization process and complicated adjustments of parameters. In this model, the singular value decomposition method is applied to reduce the dimension of complex analytical data, in order to determine source components and contributions [35]. Source components refer to the number and types of sources, and source contributions refer to the contributions of each source to each sample. The equation is as follows:

(6) $$C_{ij}=\sum_{k=1}^{m}U_{jk}D_{ik}+S$$

where: $C_{ij}$ is the content of the *j*th potential toxic element of the *i*th sample; $U_{jk}$ is the mass fraction of the *j*th potential toxic element in source *k*, representing the component of source; $D_{ik}$ is the total amount of source *k* in the *i*th sample, representing the contribution of source; *S* is the standard deviation of each source’s component. 

## 3. Results

### 3.1. Descriptive Statistics of Potential Toxic Elements in Soil

The minimum (Min), maximum (Max), mean, standard deviation (SD), coefficient of variation (CV), skewness and kurtosis of potential toxic element contents in soil are described (Table 1).

The content range of Cd (0.03~1.84 mg/kg) was the smallest, whereas that of Zn (34.3–714 mg/kg) was the largest. The contents ranges of the rest potential toxic elements were ranked in increasing order as Hg, As, Ni, Pb, Cu and Cr. The average values of As, Hg, Cr, Cd, Pb, Cu, Zn and Ni were 6.61 mg/kg, 0.29 mg/kg, 67.73 mg/kg, 0.2 mg/kg, 43.12 mg/kg, 34.77 mg/kg, 110.68 mg/kg and 29.1 mg/kg, respectively. Compared with soil background value [36], among all the potential toxic elements, the mean concentration of As did not exceed the soil background values of both Zhejiang and China, whereas Hg and Cd were opposite, namely far higher than the two background values. The mean concentration of Hg was 2.37 times the soil background value of Zhejiang and 4.46 times the soil background value of China. Correspondingly, the mean concentration of Cd was higher by 2.86 and 2.06 times. Furthermore, the mean concentrations of other potential toxic elements exceeded the soil background values to varying degrees, indicating that potential toxic elements, except As, were enriched to various extents in the study region, with Hg and Cd being the most serious. CV reflects the average variation degree of the whole sample. Thus, it can be seen that Hg in the study region showed an extreme variation, Ni and Cd showed high variations and As, Cr, Pb, Cu and Zn showed medium variations, illustrating that the data were of a high degree of dispersion. Therefore, it reflected the obvious heterogeneity of the spatial distribution of eight potential toxic elements. Moreover, the extreme variation of Hg also indicated that it was strongly subjected to human activities.

Spearman correlation analysis was used to explore the relationship among various potential toxic elements. The significant positive correlation between potential toxic elements suggests the similarity of potential toxic element sources. On the contrary, the significant negative correlation not only suggests different sources, but also may indicates a certain antagonistic effect between potential toxic elements [37]. It is obvious that significant positive correlation existed among most potential toxic elements in the study region (Table 2). The correlation coefficients of Ni-As, Ni-Hg and Ni-Cr were relatively high (r > 0.8), indicating that Ni might have the same anthropogenic source together with As, Hg and Cr. A similar situation happened between As-Cr, Pb-Hg and Cu-Zn (r > 0.7), suggesting the same agricultural or industrial sources. In addition, soil pH is one of the important factors to control the effectiveness of potential toxic elements in soil, in the way of affecting the activity and availability of soil potential toxic elements [38]. There was a certain correlation between soil pH and potential toxic element contents in the study region. To be specific, Hg, Cd, Pb and Zn were significantly negatively correlated with soil pH, whereas the remaining potential toxic elements were significantly positively correlated with soil pH, which indicated that the acidic soil in the study region had a certain impact on the concentration of potential toxic elements.

### 3.2. Ecological Risk of Soil Potential Toxic Elements

Except for Hg and Cd, the ecological risks of other potential toxic elements were slight (Table 3). According to the ecological risks, the orders of the mean values of potential toxic elements were Hg (134.08) > Cd (84.35) > Cu (9.88) > Pb (9.88) > As (7.19) > Ni (5.91) > Cr (2.56) > Zn (1.57). A slight risk posed by Hg affected over 20% samples, which was the same as mild and moderate risks posed by Hg. Furthermore, 14.92% and 10.24% of samples were at severe and even extreme severe risk levels. A total of 49.83% and 44.22% of samples showed mild and moderate risk of Cd, but 2.63% and 0.24% of samples remained at severe and extreme severe risk of Cd. The rest of the potential toxic elements posed a slight or mild risk in samples. Overall, there was relatively serious risk of Hg and Cd pollution in the study region, which corroborated the conclusion drawn from the descriptive statistical analysis. Moreover, the comprehensive ecological risk in the study region was mainly at moderate risk, with 4.24%, 20.97%, 47.29% and 27.50% of the samples being at severe, moderate, mild and slight risk levels, respectively.

**Figure 3:**
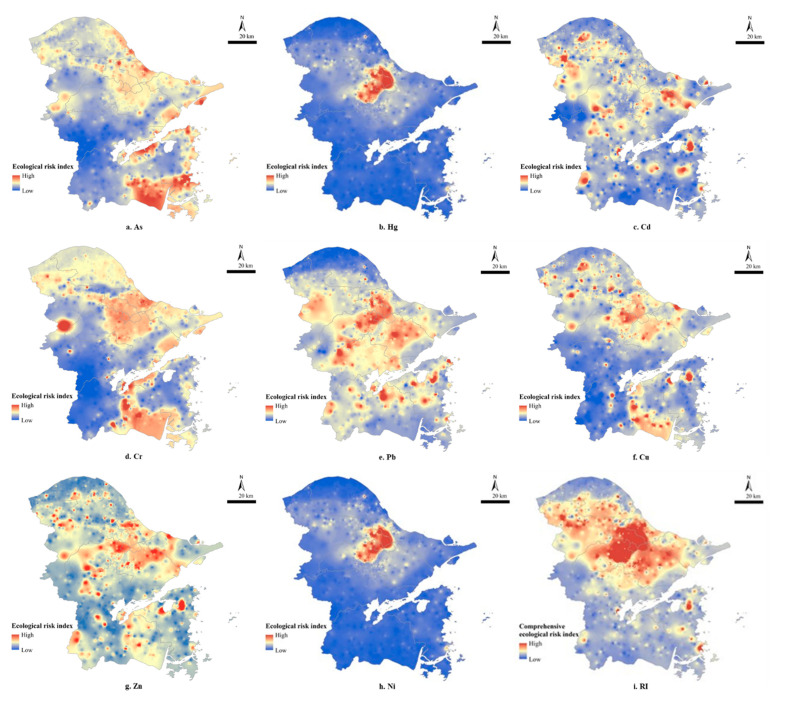
Spatial distribution of ecological risk index.

Spatially, Hg and Ni had prominently high ecological risks in urban areas, especially in the northern part (Figure 3). Cr, Pb, Cu and Zn also had high ecological risks in downtowns with larger areas. In the south of the study region, Cr had a large continuous area of high ecological risk, whereas Pb, Cu and Zn had visibly scattered dot areas. The ecological risks of As and Cd were different. The high ecological risk area of As fell in the southern coastal corner rather than the urban areas, and Cd had ubiquitous dot areas of high ecological risks across the study region. In terms of the comprehensive ecological risk, only the north, west and southwest parts of the study region were at low ecological risk, illustrating the rest of the areas were at high ecological risk levels of soil heavy metal pollution. Notably, the central urban part of the study region was subjected to moderate or even extreme severe ecological risk, indicating that the situation of heavy metal pollution was worth attention.

### 3.3. Potential Pollution Sources of Potential Toxic Elements in Soil

#### 3.3.1. Source Apportionment Based on PMF Model

**Figure 4:**
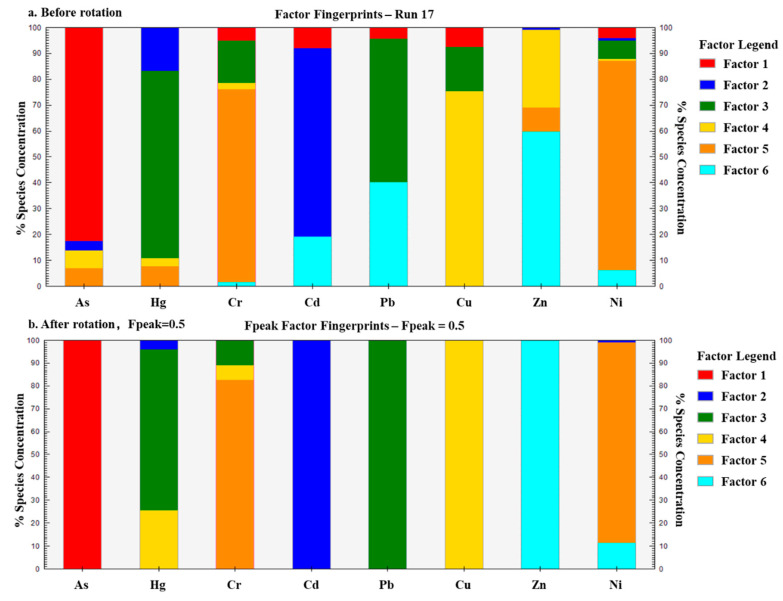
Source composition of the PMF model.

To set the number of factors as 3–9 and the operation times as 20, the initial points were randomly selected to run the PMF model successively [39]. When the number of factors was 6, the ratio of Q_robust_ to Q_true_ tended to converge and reached the minimum, and the residual error ranged from −3 to 3. Moreover, the r^2^ values of the most potential toxic elements were larger than 0.88, except that the r^2^ value of Hg was 0.4. Consequently, the six factors were able to fully explain the information contained in the original data, indicating that the six-factor source scheme was the most stable based on the PMF model (Figure 4).

Based on the PMF model, six factor components and corresponding contributions of potential toxic elements were identified (Table 4). Since the load of Factor 1 only had As, Factor 1 was obviously the main source of As. Although Cd, Hg and Ni all had loads in Factor 2, whereas the load of Cd reached up to 100%, Factor 2 was the main source of Cd. Hg, Cr and Pb all had loads in Factor 3. Since the loads of both Hg and Pb were far higher than the load of Cr (the load of Pb even reached up to 100%), Factor 3 was taken as the main source of Hg and Pb. As for Factor 4, the load of Cu reached up to 100%, which was much higher than the loads of both Hg and Cr, and it could be considered as the main source of Cu. Factor 5 had similar loads of Cr and Ni, i.e., 82.750% and 87.644%, respectively, which was the main source of Cr and Ni. Since Factor 6 had distinct loads of Zn (100%) and Ni (11.415%), it was evidently the main source of Zn.

**Figure 5:**
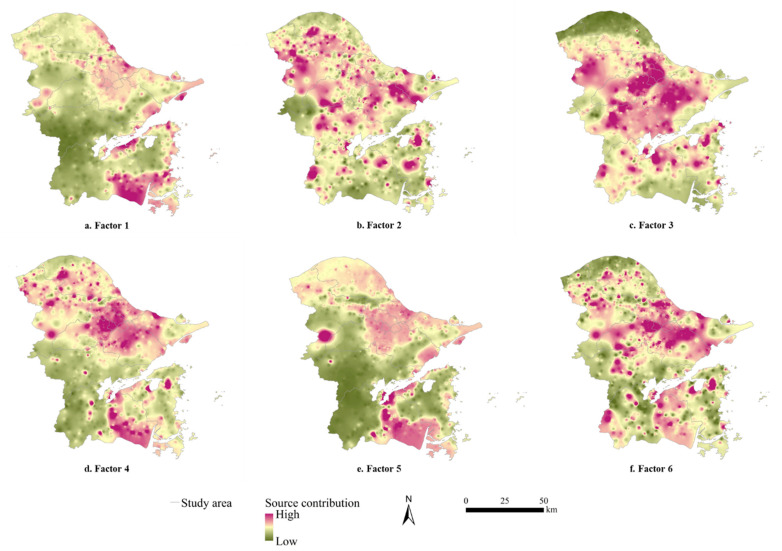
Spatial distribution of the source contribution of each sample for six factors from the PMF model.

The highest load of Factor 1 is As. Up to 99.95% of the samples are not contaminated by As (Table 3), indicating little interference of human activity. Soil parent material is the main source of As background in soil, and the content of As will gradually enrich with later development of the soil. Generally, the concentration of As is less than 15 mg/kg, whereas there were 6 samples in the study region whose concentrations of As exceeded 15 mg/kg. These samples were distributed in coastal cultivated lands from the east to the south (Figure 5a), suggesting a connection with inappropriate agricultural inputs (e.g., fertilizers and pesticides) [40,41]. Therefore, Factor 1 was a composite source of nature and agriculture.

Cd showed a high load in Factor 2. Since Cd was largely affected by external pollution, and the areas of high source contribution were mostly adjacent to the main roads (Figure 5b), Cd pollution in the study region was related to traffic emissions. Automobile exhaust can accumulate Cd in soil through atmospheric deposition and air dust adsorption [42]. Furthermore, Cd hiding in automobile tires and fuels can enter the soil through the wear and tear of automobile tires [43]. Therefore, Factor 2 was a single source of traffic emissions.

Factor 3 was principally composed of Hg and Pb, both of which concentratedly polluted the central urban region. It is obvious that Hg reached extreme variation (Table 1), and Hg and Pb had homology (Table 4). In the study region, the majority of industrial enterprises gathered in the central and north (Figure 5c). Since metallurgy, energy and chemical industries take coals as the primary fuel, the production process makes great contributions to the accumulation and enrichment of Hg. Hence, coal mining and combustion are regarded as the main source of Hg pollution in soil. The potential toxic element Hg is dispersed to the atmosphere during combustion and then enters the soil through atmospheric deposition [40]. Likewise, Pb accumulation is also largely caused by emissions of coal combustion, and gasoline combustion and wear of automobile parts can increase the concentration of Pb in soil [44,45]. Therefore, Factor 3 was a composite source of industrial discharge and traffic emissions.

The load of Factor 4 was mainly Cu. The large areas with Cu pollution had a dispersed distribution. Since the samples in this study were mostly collected from agricultural soil, unreasonable agricultural inputs might be the reason for Factor 4. As an industrially developed city, the deepening of urbanization drove more rural lands into urban construction lands, leaving limited cultivated lands to produce more crops, which aggravates the burden of cultivated lands and leads to excessive fertilizer application. The use of compound fertilizers also causes excessive accumulation of potential toxic elements (e.g., Cu and Zn) in soil [46]. The high source contribution of Factor 4 was aggregated in the northern part of the study region (Figure 5d), where the waste gas released by mechanical and electronic enterprises increases Cu pollution in soil through atmospheric deposition. Therefore, Factor 4 was a single source of agriculture.

Factor 5 was primarily composed of Cr and Ni. There was a significant positive correlation between Cr and Ni, indicating that they were polluted by the same sources (Table 3). Both Cr pollution and Ni pollution in the study region were quite slight, thus they were mainly affected by soil parent material. Since polluted Cr and Ni samples were mainly concentrated in the southern part, where a certain number of textile enterprises are distributed (Figure 5e), the waste gas settlement caused by industrial activities causes Cr accumulation [47]. Therefore, Factor 5 was a composite source of nature and industry.

The main load of Factor 6 was Zn, whose pollution did not happen in most areas. Thus, soil parent material and natural weathering processes might partly explain Factor 6. In addition, the collected soil samples were mainly distributed around roads and mechatronics enterprises. The exhausts of automobiles and electroplate factories inevitably lead to the enrichment of Zn [48]. Therefore, Factor 6 was a composite source of nature, industrial discharge and traffic emissions.

#### 3.3.2. Source Apportionment Based on Unmix Model

**Figure 6:**
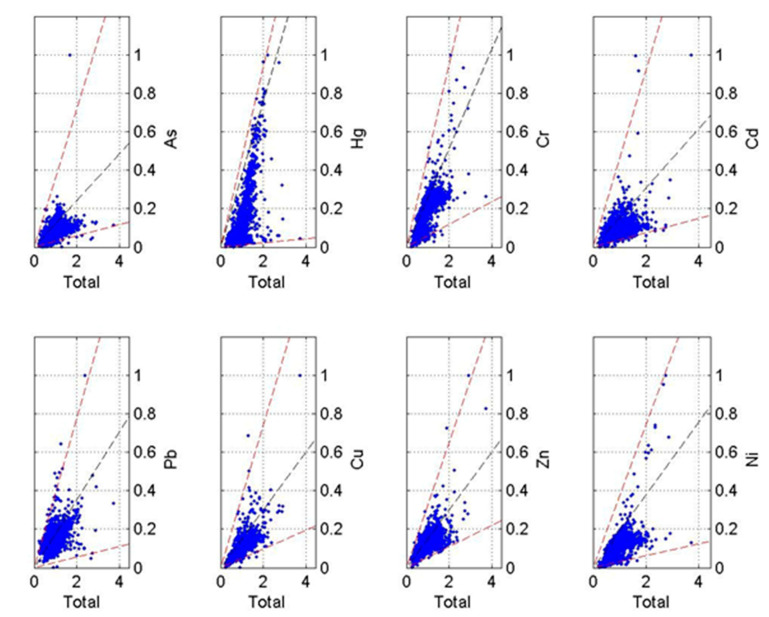
Geometric distributions of eight potential toxic element contents (after normalization).

The normalization of original data was processed to eliminate the influence of the content difference among eight potential toxic elements. Since the boundary (i.e., red virtual line) appeared in all data boxes of eight potential toxic elements (Figure 6), rational boundaries were considered to exist among all eight potential toxic elements. Thus, no data needed to be removed and all of them could be used in the Unmix model. When the number of sources was set to six, the Min Rsp was 0.97 and the Min Sig/Noise was 2.77 (Table 5), both of which were greater than system requirements (i.e., Min Rsq > 0.8 and Min Sig/Noise > 2). Consequently, the result that 97% of the variance of species could be explained was stable and optimal, indicating the six-source scheme was the most reliable based on the Unmix model.

Based on the Unmix model, six source concentrations of potential toxic elements after normalization were identified (Table 5). All kinds of potential toxic elements have loads in Source 1, especially Hg, Cr and Pb. The contribution of Source 1 to Hg was the largest (peaking at 94%), which was far higher than Cr (23%) and Pb (28%). Hence, the characteristic element of Source 1 is Hg. Pb and Zn had relatively higher loads in Source 2, whose contributions were 47% and 21%, respectively. Whereas for Zn, the contribution of Source 6 was higher than that of Source 2. Hence, the characteristic element of Source 2 is Pb. Source 3 had relatively higher loads of Cu and Zn. The contribution of Source 3 to Cu is 29%, which was lower than that of Source 6 (39%). The contribution of Source 3 to Zn is 18%, which was lower than that of Source 6 (29%) and Source 2 (21%). Hence, the characteristic elements of Source 3 were considered to be Cu and Zn. The load of Cd in Source 4 was much higher than that of other potential toxic elements, and the contribution of Source 4 to Cd was the largest, reaching up to 56%. Hence, Cd was mainly affected by Source 5. In addition to Hg and Cd, all other potential toxic elements had certain loads in Source 5. The contribution of Source 5 to As was 52%, which obviously exceeded that of Source 1 (20%) and Source 6 (22%). Hence, the characteristic element of Source 5 was As. Similar to Source 1, all eight potential toxic elements had loads in Source 6, particularly Cr and Ni. The contributions of Source 6 to Cr and Ni were 61% and 76%, respectively, which were the highest among all sources. Hence, Source 6 was regarded as the main source of Cr and Ni.

**Figure 7:**
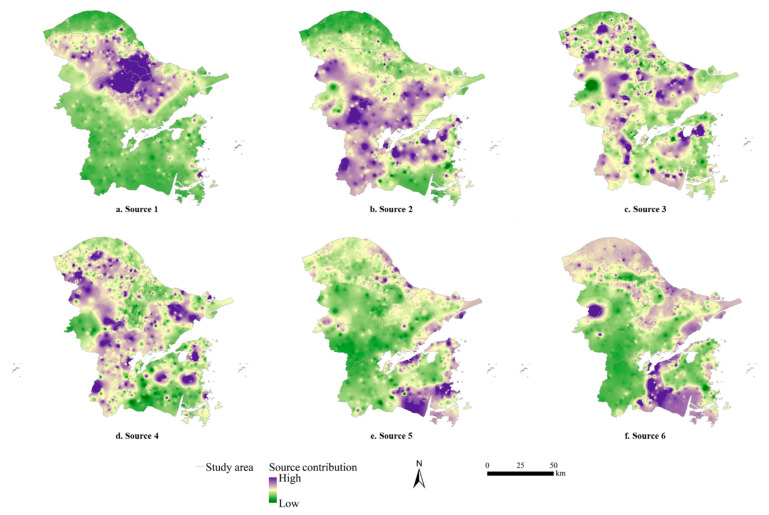
Spatial distribution of the source contribution of each sample for six factors from the Unmix model.

Source 1 had a predominate load of Hg. The high-source contribution area of Source 1 was distributed in contiguous urban areas, and scattered across northern and southern sides and the southeast coastal dot area (Figure 7a). Source 1 was mainly affected by industrial discharge (wastewaters, exhaust gas and waste residue) caused by enterprises or factories, which was similar to the results based on the PMF model. Conversely, the contribution of Source 1 to Pb was lower, based on the UNMIX model, making the component of Source 1 simpler. Therefore, Source 1 was a single source of industrial discharge.

Source 2 was characterized by high loadings of Pb. The CV of the measured concentrations of Pb was 37.22%, suggesting limited human disturbance. The source contributions in the southwest of the study region was higher than that in the eastern coastal area (Figure 7b), which was similar to the spatial distribution of soil pH, namely higher soil pH in the coastal area but continuously reduced soil pH in the western area close to inlands. Pb in acidic soils dissolves continuously with the increasing of soil acidity [49], indicating that lower soil pH contributes to higher Pb content in soil. Therefore, Source 2 was a single source of nature affected by soil pH and soil formation process.

Source 3 was heavily loaded with Cu and Zn. There were many dot areas with high source contributions in the study region especially the northern part (Figure 7c). And a significant positive correlation also showed between Cu and Zn (Table 2). Electroplate wastewater can bring about excessive Cu and Zn in agricultural soils through irrigation [50]. The accumulation of Cu and Zn are also closely related to the harmful discharge of mechanical and electronic enterprises in the study region. Therefore, Source 3 was a single source of industrial discharge.

The characteristic element of Source 4 was Cd, whose spatial distribution of source contribution was almost the same as that obtained based on the PMF model. Therefore, Source 4 was still considered as the single source of traffic emissions generated by automobile exhausts and wear of automobile parts.

As accounted for, the highest load was in Source 5. The source contributions of As were higher in coastal areas but lower in inland areas. Therefore, similar to the results based on PMF model, Source 5 was a single source of nature affected mainly by soil parent material.

The characteristic elements of Source 6 were Cr and Ni, and were consistent with Factor 5 based on the PMF model. Therefore, Source 6 was a composite source consisting of soil parent material derived from nature and industrial discharge caused by human activities.

## 4. Discussion

### 4.1. Comparison of the PMF Model and the Unmix Model

#### 4.1.1. Model Evaluation

To evaluate the applicability of the PMF and Unmix models, the regression coefficient and residual were used to estimate the fitting goodness of the model (Table 6). For the regression coefficient, the closer the r^2^ and slope are to 1 and the intercept is to 0, the better the fitting effect is. For the residual, the closer the mean is to 0 and the smaller the range is, the better the fitting effect is.

The fitting effect of Hg in the UNMIX model (r^2^ = 0.999) was better than the PMF model (r^2^ = 0.399), which might be related to the large quantities of polluted samples, high variation coefficient and great fluctuation of original Hg samples. Although Hg pollution was mainly caused by industrial discharge, the specific sources were probably more diverse and complex, which needs further exploration in future works. The fitting effects of Pb, Cd and Zn in the PMF model were satisfactory, with r^2^ all exceeding 0.97. It is generally considered that the fitting goodness of model being over 0.8 is relatively high. Although the r^2^ of Cr was slightly lower than other potential toxic elements (0.8834), the fitting goodness was satisfactory. Similarly, although the r^2^ of Zn was 0.8406 in the UNMIX model, the fitting goodness was satisfactory. The r^2^ of other potential toxic elements (i.e., Cd, As and Ni) were even all greater than 0.97. In addition, the residuals of the two models ranged from −3 to +3. Moreover, the range of the UNMIX model appeared smaller, falling between −1 and +1, which indicated that the fitting effect of the UNMIX model was better to some extent.

#### 4.1.2. Source Components and Contributions

Both the PMF model and the Unmix model identified six sources that could be categorized into anthropogenic and natural sources (Table 7). The former included industrial discharge, traffic emissions and agricultural inputs, and the latter included soil parent material and soil formation processes. Although composite sources existed in source apportionment based on both models, the source components composition was not completely consistent. Overall, industrial discharge was the main source heavy metal pollution in the study region, and the influence of anthropogenic sources on the study region was more significant than that of natural sources.

The most polluted potential toxic elements, Hg and Cd, had a consistent pollution source in two models. Hg pollution was mainly caused by three industrial wastes of enterprises or factories. Cd pollution was related to traffic emissions such as automobile exhausts, wear and settlement of automobile parts. Both of the models showed that the pollution source of As was mainly soil parent material. However, the PMF model also apportioned the pollution source of inappropriate agricultural inputs. Due to the high fitting goodness of As in the PMF model (r^2^ = 0.9128), as well as the existence of a few slightly polluted samples in the study region, it was necessary to consider the input of fertilizers and pesticides as the source of As pollution. The difference between the source apportionment results of Cr and Ni was whether they were affected by soil parent material source, except for industrial sources. Since the UNMIX model had a higher goodness of fit for Cr and Ni, as well as their CV reached moderate and severe variations respectively, Cr and Ni pollution were more subject to human interference. Therefore, the pollution source apportioned by the UNMIX model was more reasonable, namely a single source of industrial discharge. In terms of Cu and Zn, they had their own pollution source in the PMF model, but they were characteristic elements of Source 3. These two potential toxic elements showed an extremely significant positive correlation at the 0.01 level, indicating that they were very likely to have similar or even the same sources. According to the goodness of fit for Cu and Zn, the PMF model appeared better. Thus, the pollution source apportioned by the PMF model was more referential, namely composite sources of soil parent material, industrial discharge and traffic emissions. The source contribution of Pb distributed variously based on the PMF model and the UNMIX model, whose main difference was whether high contributions aggregated in urban areas. Pb had a high ecological risk in the central urban area, which might attribute to the high-density enterprise distribution there. Therefore, Pb pollution was closely related to not only natural sources (e.g., soil pH) but also industrial sources (e.g., industrial wastes discharge).

### 4.2. Comparison on Source Apportionment Studies

Nature, agricultural inputs, industrial discharge and traffic emissions are common pollution sources of potential toxic elements in soil. Since the Yangtze River Delta of China is a typical industrial region that has been plagued by heavy metal pollution for a long time, many researchers were fond of conducting source apportionment studies there. In the same case study area, Shao et al. applied the principal component analysis (PCA) and the finite mixture distribution model (FMDM) to identify and apportion the pollution sources of seven soil potential toxic elements (i.e., Cr, Cd, Hg, Cu, Zn, Ni and As) [51]. It was found that Cr, As and Ni were primarily from natural source of soil parent material; Cd, Cu and Zn were primarily from composite sources (e.g., traffic pollutants, household garbage and agricultural inputs); and Hg was primarily from industrial discharge. Although this study identified three sources, that comprised half of our study, both studies proved that the industrial source was main pollution source of soil potential toxic elements. Jia et al. further explored the different effects of polluting enterprise types on Cd and Hg, which were the most polluted potential toxic elements and were significantly affected by human activities in the Yangtze River Delta of China [2]. This study divided enterprises into four categories, i.e., textile industry, chemical industry, metalwork industry and other industry. It was revealed that different industry classes leading to soil pollution varied from both areas and in potential toxic element types. Soil Cd pollution was chiefly affected by excessive fertilization and coal mining, and the metalwork industry was the most seriously Cd-polluted industry, whereas Hg pollution in soil was closely related to enterprise pollution, on which the chemical industry had the most influence.

## 5. Conclusions

As an economically developed area, the eastern coast of China is highly industrialized and urbanized. However, widespread soil pollution has become more and more serious in eastern industrial cities. In this study, an industrialized city on the eastern coast of China was taken as the study region, and the PMF and Unmix models were adopted to explore the source identification and apportionment of potential toxic elements in soil. The results showed that anthropogenic sources exerted a more significant impact on soil heavy metal pollution than natural sources. Traffic exhausts and automobile wear, industrial wastes discharge and excessive fertilizer and pesticide inputs were main sources of anthropogenic sources. Soil parent material and soil formation process were typical natural sources. In the study region, the enrichment of As in soil was subjected to soil parent material, part of which was also affected by inappropriate agricultural inputs. The pollution of Hg and Ni as well as the enrichment of Cr in soil were mainly caused by industrial discharge from enterprises and factories. The Cd pollution in soil was related to traffic emissions, such as automobile exhausts and wear and settlement of automobile parts. The sources of Pb pollution in soil were related to not only natural sources (e.g., soil pH) but also industrial sources (e.g., industrial wastes discharge).

## Figures and Tables

**Table 1:** Descriptive statistics of potential toxic element contents in soil in the study region (*n* = 2051).

Potential Toxic Elements	As	Hg	Cr	Cd	Pb	Cu	Zn	Ni
Min	0.868	0.015	6.04	0.03	8.13	4.28	34.3	2.89
Max	69.8	2.26	326	1.84	263	315	714	234
Mean	6.61	0.29	67.73	0.2	43.12	34.77	110.68	29.1
SD	2.99	0.3	29.4	0.1	16.05	16.69	36.52	15.59
CV	45.23	103.45	43.41	50.00	37.22	48.00	33.00	53.57
Skewness	5.11	2.34	1.47	7.26	2.44	4.56	5.16	4.14
Kurtosis	97.29	6.71	10.23	101.37	21.62	51.17	62.5	41.52
SB values of Zhejiang	9.2	0.086	52.9	0.07	23.7	17.6	70.6	24.6
SB values of China	11.2	0.065	61	0.097	26	22.6	74.2	26.9

Note: The unit of CV was % and units of others were mg/kg. SB represented soil background [35].

**Table 2:** Correlation analysis of soil potential toxic element contents and pH in the study region.

r	As	Hg	Cr	Cd	Pb	Cu	Zn	Ni	pH
As	1								
Hg	0.018	1							
Cr	0.753 **	0.302 **	1						
Cd	−0.014	0.385 **	0.045 *	1					
Pb	−0.057 *	0.712 **	0.157 **	0.444 **	1				
Cu	0.564 **	0.445 **	0.753 **	0.357 **	0.345 **	1			
Zn	0.417 **	0.373 **	0.571 **	0.494 **	0.528 **	0.716 **	1		
Ni	0.813 **	0.094 **	0.923 **	−0.006	−0.04	0.690 **	0.512 **	1	
pH	0.346 **	−0.412 **	0.210 **	−0.051 *	−0.554 **	0.081 **	−0.050 *	0.372 **	1

* Correlation is significant at the 0.05 level. ** Correlation is significant at the 0.01 level.

**Table 3:** Ecological risk index of soil potential toxic elements in study region.

Ecological Risk Index	Potential Toxic Elements	Min	Max	Mean	Proportion of Ecological Risk Level				
					Slight	Mild	Moderate	Severe	Extreme Severe
$E_{r}^{i}$	As	0.94	75.87	7.19	99.95	0.05	0.00	0.00	0.00
	Hg	6.98	1051.16	134.08	23.31	24.57	26.96	14.92	10.24
	Cr	0.23	12.33	2.56	100	0.00	0.00	0.00	0.00
	Cd	12.86	788.57	84.35	3.07	49.83	44.22	2.63	0.24
	Pb	1.72	55.49	9.10	99.95	0.05	0.00	0.00	0.00
	Cu	1.22	89.49	9.88	99.85	0.10	0.05	0.00	0.00
	Zn	0.49	10.11	1.57	100	0.00	0.00	0.00	0.00
	Ni	0.59	47.56	5.91	99.90	0.10	0.00	0.00	0.00
RI		49.70	1201.72	254.64	27.50	47.29	20.97	4.24	-

Note: Min, Max and Mean had no units, and the unit of proportion of ecological risk level was %.

**Table 4:** Source components and contributions of potential toxic elements based on the PMF model.

	Species	Factor 1	Factor 2	Factor 3	Factor 4	Factor 5	Factor 6
Source components (mg/kg)	As	6.589	0	0	0	0	0
	Hg	0	4.000	0.073	0.027	0	0
	Cr	0	0	7.428	4.165	55.609	0
	Cd	0	0.196	0	0	0	0
	Pb	0	0	43.032	0	0	0
	Cu	0	0	0	34.509	0	0
	Zn	0	0	0	0	0	110.390
	Ni	0	0.272	0	0	25.306	3.296
Source contributions (%)	As	100	0	0	0	0	0
	Hg	0	3.877	70.452	25.671	0	0
	Cr	0	0	11.053	6.198	82.750	0
	Cd	0	100	0	0	0	0
	Pb	0	0	100	0	0	0
	Cu	0	0	0	100	0	0
	Zn	0	0	0	0	0	100
	Ni	0	0.942	0	0	87.644	11.415

**Table 5:** Source concentrations of potential toxic elements based on the Unmix model (Normalized).

Species	Source 1	Source 2	Source 3	Source 4	Source 5	Source 6
As	0.059	0.039	−0.022	0.016	0.439	0.056
Hg	0.398	−0.002	0.022	0.044	0.006	0.007
Cr	0.153	0.095	0.049	−0.008	0.177	0.358
Cd	0.045	0.042	0.082	0.732	0.027	0.047
Pb	0.132	0.617	−0.019	0.091	0.125	0.049
Cu	0.080	−0.015	0.503	0.039	0.058	0.117
Zn	0.063	0.229	0.364	0.110	0.102	0.100
Ni	0.070	−0.005	0.022	−0.024	0.066	0.267
Total	0.289	0.104	0.057	0.071	0.100	0.330
9 Species, 2051 Obs., 6 Sources,						
Min Rsq = 0.97, Min Sig/Noise = 2.77						

Note: Due to the error of data uncertainty, the concentrations of source components of some species appeared negative. After inspection, the errors above were acceptable.

**Table 6:** Goodness of fit of the PMF model and the UNMIX model.

Goodness of Fit	Model	Parameter	As	Hg	Cr	Cd	Pb	Cu	Zn	Ni
Regression coefficient	PMF	r^2^	0.913	0.399	0.883	0.970	0.982	0.948	0.981	0.931
		Slope	0.993	0.085	0.842	0.015	2.557	5.089	9.698	3.913
		Intercept	0.845	0.067	0.980	0.923	0.938	0.846	0.910	0.858
	UNMIX	r^2^	0.998	0.999	0.925	1.000	0.960	0.949	0.841	0.972
		Slope	1.000	1.002	0.982	0.998	0.973	0.977	1.068	1.018
		Intercept	−0.001	−0.001	0.004	0.000	0.003	0.002	−0.009	−0.005
Residual	PMF	Mean	0.000	0.090	0.012	−0.001	0.001	0.001	0.001	0.000
		Min	−0.063	−0.666	−2.187	−0.027	−0.325	−0.396	−0.440	−0.536
		Max	0.247	0.336	1.857	0.097	1.178	1.081	1.154	1.096
	UNMIX	Mean	0.001	0.000	0.000	0.000	0.000	0.001	0.001	0.003
		Min	−0.011	−0.050	−0.452	−0.010	−0.107	−0.122	−0.365	−0.133
		Max	0.037	0.030	0.294	0.017	0.225	0.218	0.218	0.202

**Table 7:** Source components and contributions based on the PMF model and the Unmix model.

Model	Components	Identification	Contributions
PMF	Factor 1	Composite source of soil parent material and agricultural inputs	12.50%
	Factor 2	Single source of traffic emissions	13.10%
	Factor 3	Composite source of industrial discharge and traffic emissions	22.69%
	Factor 4	Single source of agricultural inputs	16.48%
	Factor 5	Composite source of nature and industry	21.30%
	Factor 6	Composite source of nature, industrial discharge and traffic emissions	13.93%
Unmix	Source 1	Industrial discharge source	30.39%
	Source 2	Natural source of soil pH and soil formation forming process	10.94%
	Source 3	Industrial discharge source	5.99%
	Source 4	Traffic emissions source	7.47%
	Source 5	Natural source of soil parent material	10.52%
	Source 6	Natural source of soil parent material and industrial discharge source of human activities	34.70%

## Data Availability

Not applicable.
